# Terrorist Attacks: Do We Know How to Assess the Results?

**DOI:** 10.1100/tsw.2001.319

**Published:** 2001-10-24

**Authors:** William J. Manning

**Affiliations:** University of Massachusetts, Amherst, MA, USA

**Keywords:** terrorism, air quality, human health, effects assessment, toxicology

## Abstract

On September 11, 2001, terrorists destroyed the World Trade Center (WTC) in New York City. Explosions and fires resulted in the complete collapse of the two WTC towers. The collapsing towers served as enormous point sources of gaseous and particulate air pollution, seen as huge plumes of smoke and dust. The smoke contained volatile organic compounds and fine particles and aerosols. The dust fraction contained parts of ceiling tiles, carpets, concrete, adhesives, asbestos, chromium, lead, titanium, and many other elements and materials. Whether there were unusually toxic ingredients in the plumes is largely unknown.

Immediate questions raised included: (1) what had been released and how was it distributed, (2) what were the short- and long-term health effects and risks for survivors and rescuers, and (3) what methods should be used to assess the results of terrorist attacks.

An earlier terrorist attack on the WTC had prompted the National Institute of Environmental Health Sciences (NIEHS) to fund researchers at several east coast universities to develop an approach to sample air quality after attacks. As a result, they were able to begin limited air and dust sampling on September 12, using standard methods. Their initial results indicated that what they monitored did not pose short- or long-term health risks, even for asbestos. Whether or not these methods were appropriate for assessing the aftermath of the collapse of huge buildings is unknown. Perhaps the NIEHS needs to develop specific guidelines for assessing the results of such disasters.

The short-term health effects for survivors and rescuers seem to be eye and throat irritation, and excess mucus in noses and throats due to pulmonary system clearing of noninhalable particles above 2.5 microns (μ) in diameter. Rescuers were also exposed to considerable amounts of volatile organic compounds and oxides of nitrogen from exhausts of portable electric power generators and diesel-powered trucks, and debris removal equipment.

Long-term health effects may be due to inhalation of particles smaller than 2.5 μ that become lodged in alveoli in lungs. For example, the force of the collapse of the towers may have caused the release of asbestos particles at or below 2.5 μ in diameter. Air quality monitoring for asbestos seems to have been done for particles of 5.0 μ in diameter, in compliance with EPA standards for asbestos.

Terrorists have many chemical and biological weapons available to them. Examples were given in a report published by the General Accounting Office in 1999. Chemical weapons include choking agents (chlorine, phosgene), nerve poisons (sarin, tabun), blood poisons (hydrogen cyanide), and biological weapons, such as anthrax and viruses (Ebola and smallpox). Sarin was used by the Japanese terrorist group Aum Shinrikyo to kill or sicken people in Tokyo subways in 1995.

Bioterrorists are curently mailing anthrax spores in or on envelopes to people in various parts of the country. One person has died and others have developed cutaneous infections. Treatment with antibiotics seems effective, but few physicians are familiar with anthrax and incidence may be overlooked. Rapid diagnostic tests are needed. Tracing various strains of *Bacillus anthracis* to their origins also seems to be difficult.

Unlike the Luddites who tried to halt the industrial revolution in England, today's terrorists are worldwide in distribution and will not be easily eliminated. We are beginning to understand that living with terrorism has become part of our lives. It is imperative that we learn how to quickly assess the short-term effects of terrorist attacks and determine long-term health risks.

For our own protection, we need to develop standard adequate assessment methods. It is disconcerting to realize that government agencies, university researchers, and private consulting companies look for different things and use different standards and measurements for the same things. People need to be trained differently and we need to consider enlarging the scope of assessments. Perhaps terrorist attack response teams need to be developed?

## References

*Nature* **413**, 335 (2001) www.Nature.com
New Jersey Assessment Program www.eohsi.rutgers.edu/rc/respons.shtml
*The New York Times*, Science Times 2 & 16 October (2001)
